# Theoretical Considerations of Pulmonary Percussion Notes

**Published:** 1909-09

**Authors:** George Hely-Hutchinson Almond


REFERENCES.

### THEORETICAL CONSIDERATIONS OF PULMONARY PERCUSSION NOTES.

BY

GEORGE HELY-HUTCHINSON ALMOND, M.A., M.B. (Oxon.).

THE subject may best be prefaced by a brief consideration of a few characteristics of sound production, especially of those that apply to the percussion of a membrane.

When a drum is struck a certain note is produced, and the four cardinal characteristics of this note are pitch, intensity, quality and duration.

The *pitch* depends upon: (*a*) The superficial tension—the tenser the membrane the higher the pitch; (*b*) the thickness of the membrane—the thicker the membrane the lower the pitch; (*c*) the area of surface—the larger the area the lower the pitch. In other words, the pitch varies directly with the tension and inversely with the thickness and surface area.

The *intensity* depends upon the amplitude of the vibration, and is thus directly proportional to the force of the blow.

The *quality* depends on the additional tones or overtones that accompany the chief tone. They vary in any given drum according to the part struck.

We must also include under this category the noise—or in other words, the irregular vibrations set up by the beat. It is by the quality of a note that we are enabled to recognise the instrument on which a given note is played, *e.g.* fiddle, flute, oboe, etc.

The *duration* varies directly with the intensity, and inversely with the pitch. It is also determined by any damping effect, such as the continued contact of the drumstick after the beat, or by the contact of a layer of flannel placed on the surface ( = muffling).

We must clearly understand that the pitch of a drum note depends entirely on the condition of the membrane, and that it is not influenced in any degree by the presence of any cavity, such as we get in an ordinary military kettle-drum with its two surfaces, nor yet by the hemispherical metal resonator that we get in a tympanum.

It is no small task to compare a drum beat to a pulmonary percussion note. In the first place, the thoracic membrane is not homogeneous. The stretched, taut portion of the thoracic wall, which causes the thorax to retain its shape even after evisceration, consists mainly of the ribs, sternum and intercostal cartilages, and the intercostal muscles together with the posterior intercostal membranes. This we shall look upon as our thoracic membrane. Its shape, moreover, is irregular, as are its attachments. On the surface are the skin and the subcutaneous tissues, the scapula and various muscles, which act as damping elements; whilst of the contents of the chest, some are solids, some are vesicular, some are hollow, some are central, whilst others are in contact with the wall.

The method of percussion in vogue is to use the index or middle finger of the left hand as pleximeter, and one or more of the fingers of the right hand, usually the middle or the index and middle, as plexor. The object of the pleximeter is to counteract the muffling effect caused by the covering tissues.

The detection of the actual pitch of a given percussion note would be an almost herculean task, but for our present purpose a comparison of the notes obtained on chests of different sizes will suffice. If we were to set before us a series of healthy persons—say, an infant, a child of six, a youth of 14, and an adult—and were to percuss a corresponding area of chest wall of each, we should obtain a series of notes, highest on the infant and lowest on the adult. This we should expect, for the chest wall of the smaller person is both of a smaller area and thinner than the larger. In the adult the tension, being greater than in the youth or child, would by itself cause an inverse ratio of pitch, but its effects are masked by the two preceding qualities.

As the duration of the note varies inversely with the pitch, we would expect it to be more prolonged in the adult, and this we should find to be the case. That the intensity varies directly with the force of the blow can be very easily demonstrated, and this need not be further discussed here.

If we refer again to our series of chests, we should observe, as we percussed each chest in turn, that the note produced was that of normal pulmonary resonance. If, however, we had produced on the adult a note identical with that produced on the infant, we should have termed it high pitched, and, *vice versa*, we should have termed a note such as we had produced on the adult low pitched if we had obtained it on the infant. Normal resonance is, then, a relative term. It denotes a certain pitch for a certain size of chest. But the note is not quite uniform in either pitch or quality over the different areas. Certain portions of the chest also are muffled by underlying fat, thick muscles or bone (scapula), and here we have an added quality of thud, whilst both the intensity and the duration of the note are lessened. Percussion over a rib or over the sternum by the addition of the osteal element slightly alters the quality of the note ; and, lastly, the presence of solid organs more or less subjacent produces, as we shall see, its own variation. Yet the note, wherever we get it, we term one of "normal resonance."

The adjective "normal" is, then, relative, and depends upon our education. But on each chest and on each portion of chest percussed there is—provided there is no solid organ lying in contact with the chest wall—a pulmonary resonance. Apart from pitch, duration and intensity, we have a quality peculiar to the thorax. To bring this quality into further relief, let us compare our thoracic scale with a scale composed of some of the air containing abdominal viscera. The note produced by percussing each particular organ varies much, and depends largely upon the amount of dilatation, *i.e.* the size of the cavity and the tension. The larger the surface area and the lower the tension the lower will be the note, and *vice versa*. Taking these, however, into combination, it will be found that the following is a more or less reliable order : cæcum, stomach, colon, small intestine. We have in each case the parietal surface, which with the abdominal wall immediately above forms a circumscribed membrane, and this is responsible for the production of the pitch, and to a certain extent the quality. Without going into further detail, we have, then, these two separate scales—the thoracic and the visceral. The pitch varies in either in height and depth, but what essentially distinguishes them is the quality of the note.

Thus far we have assumed that the thoracic membrane is mainly responsible for the production of pitch. We have now to show, as far as we can, that we have grounds for this assumption ; and we have, moreover, to investigate how the quality of the percussion note comes to be what it is.

From a remark made by Dr. T. J. Horder, to the effect that if a cloth was laid over a corpse in a *post-mortem* room he was unable to detect by percussion whether the thoracic contents had been removed or not, we were led to perform the following experiment. Selecting two subjects whose deaths had been due to surgical-abdominal causes, we percussed corresponding portions of the chest of each previous to any incisions being made. The note in both cases was, if anything, of a slightly higher pitch than one would have expected had the subjects been alive. " Pulmonary resonance " was present to a very considerable extent, though it was not quite so marked as one would have expected in the living subject. The pitch of the one which was now to be chosen as control was slightly higher than the test case, which was now eviscerated of its thoracic contents per abdomen. An incision was at the same time made in the neck in order to cut through the structures passing up to the naso-pharynx. The empty thorax, with its two openings, was now percussed The note was tympanitic, and at the same time boxy, and there was an entire absence of pulmonary resonance.

Both openings were now completely closed by manual pressure, so that the thoracic membrane was as nearly as possible in its original posture, the abdominal contents being placed as much as possible *in situ*. The pitch of the note was now equal to that of the control, that is, it was, compared with its previously full condition, slightly raised. The quality of the note as closely resembled the normal pulmonary quality as did the note of the control, and thus of the same thorax before its evisceration. These investigations demonstrate two points: firstly, that the quality that we call " pulmonary resonance " is due in some part at least to the thorax apart from its contents ; and, secondly, that the chest wall depends upon its attachements to cause a certain tension in order to produce its note. The following experiments will, I think, demonstrate more clearly that tension, and not the presence of an enclosed cavity, was responsible in our last experiment for the production of pitch. From a number of 3-inch earthenware flower-pots we selected two which on percussion of their sides with a metal instrument yielded notes of identical pitch. They were laid on a board covered with a thin layer of cotton-wool. If both were laid rim downwards the note and the quality were identical. If one was now put right way up the notes were still of identical pitch, but the quality of the character was altered, the note being much more clear and ringing. If both pots were now put face downwards, and one of them was packed with cotton-wool, the pitch of both was still identical, but the note of the unpacked one was clearer than the other. From this we are able to conclude that the pitch was unaltered so long as the sides of the pots were enabled to vibrate comparatively freely. If one of the pots was now firmly pressed down and then percussed, the tension of the sides of the pot was considerably raised, and the pitch became very much higher. With regard to the quality of the note, we observed that it was considerably altered when one of the pots was placed rim downwards. This must be due to either the damping effect caused by the rim of the pot lying on the cotton-wool—and this damping effect is increased when the pot is packed with cotton-wool—or else it must be due to the resonance caused by the formation of a cavity. That it was not due to the latter was proved by placing one of the pots face downwards as before, and by placing another smaller pot packed tightly with damp earth inside the other, so that the smaller pot at no point touched the outer one. The quality in the note of both pots was now found to be identical, though the cavity inside the one was now only about half that inside the other.

Turning now to the percussion of lung tissue, we note that the pitch and quality vary according to the distension. A collapsed lung has a somewhat low-pitched note, and a considerable degree of resonance. As the lung is distended with air the pitch rises, and the amount of resonance diminishes. The rise in pitch is not very marked at first, but when the lung is very much distended it becomes considerably raised. This slow rise of pitch with increasing tension is retarded by a counteracting effect, which Dr. J. F. Bullar has drawn attention to. He points out that a large mass of lung (collapsed) has a lower note when percussed than half that mass. In other words, the larger the mass percussed the lower is the pitch, provided that the shape of the mass is fairly constant. It is interesting to note that a lung distended to about its normal size gives a note with a pitch very much raised and a resonance very much less marked than the note produced over the chest before evisceration.

We have already observed that the pitch has some close connection with the size of the chest wall, and that it is not altogether dependent on the presence of the lungs. In our *post-mortem* experiment it was impossible, of course, to judge that the exact former tension of the wall could be maintained by manual pressure, and this may or may not have accounted for the slight rise of pitch after evisceration. If the tension of any portion of the chest wall is in any way altered, the pitch undergoes a change. Thus, if the tension of the supra-clavicular region is increased by the forcible rotation of the head and the opposite side, the pitch of the note is considerably raised. And again, as Bullar points out, if a small tube is inserted into the pleural cavity so as to form a pneumothorax, the note is not much altered ; but if the tension in the newly-formed cavity is now raised by the pumping in of air, there is a considerable rise of pitch.

The sudden collapse of the lungs when the pleura is opened demonstrates that the tension of the chest wall is inherent, and not due to the presence of the lungs. This is also apparent from the passive movement of the lungs, as the chest wall expands and contracts during respiration. During full inspiration, too, when the thorax is more tense, the percussion note, as Dr. Reginald Thompson points out, is higher than after a full expiration.

The question that now arises is, Do the lungs by their presence in any way influence the pitch ? As we have stated above, the formation of an experimental pneumothorax scarcely alters the note so long as the pressure on both sides is that of the atmosphere. For healthy lung tissue we must conclude, then, that the influence of the lungs, if any, must be very small.

The problem bears another aspect, however, when we turn our attention to lung tissue altered by disease. Dr. Bullar quotes several experiments of his own which have some bearing upon this point. He was able to make a froth of gelatine, in which the cavities of air were about the same size and as numerous as the vesicles of the lung. Percussion of this gave similar results to his percussion of lung tissue. He made another froth with smaller and less numerous air cavities, and the pitch on percussion of this was higher mass for mass. Portions of congested lung and of pneumonic lung, which still contained some air in the vesicles, gave similar results. How are we to account for these facts? Are we to conclude that the chest wall is responsible for the pitch in health, but that in disease it is nothing more than an inert membrane? Or is it possible that congested lung in any way affects the vibrations of the chest wall? Bristowe considers that this rise of pitch is due to probable diminution of vibrating area of chest wall over part of the lung. We would prefer to review the situation in the following manner. We would regard the pitch as being mainly due to the size, structure and tension of the chest wall, modified to a certain extent by the passive resistance of the subjacent lung tissue. When the lung becomes congested, and the lung tissue is more solid, this passive resistance is increased, the resiliency of the chest wall is diminished. The tension is thus raised and likewise the pitch. When a solid body lies immediately subjacent, as, for instance, solid lung, liver or heart, the resistance is absolute, and the chest membrane is unable to vibrate at all. The note is dull, *i.e.* there is the noise of the beat, but no pitched note.[1] With regard to the deep cardiac and hepatic dullness, the explanation would be as follows: Light percussion produces vibrations of small amplitude, and there is no dulling of the note. Hard percussion, however, produces vibrations of the same pitch but of much larger amplitude, and since there is only a somewhat thin layer of passive resistance in the form of lung tissue, the solid organ underneath quickly puts an end to the vibrations, and there is a rapid dulling of the note. When a considerable portion of the lungs becomes solid the note over the resonant part of the chest is noticed to be high-pitched. This would be explained by the fact that the area of vibrating membrane is diminished.

There used to be a theory that the bronchi were responsible for the percussion note. Dr. Bullar clearly disproved this by injecting gelatine into all the larger bronchi of a sheep's lung. The lung remained resonant as before. We only quote this because at one time this theory entered largely into discussions upon the subject.

1 When fluid intervenes as in pleural effusion it is stated by Dr. Samuel West that half-an-inch is sufficient to produce absolute dulness.

The percussion note of the stomach brings up a point of difficulty at first sight. It lies partly under the abdominal wall and partly under the ribs. The percussion note and quality, especially if the organ is enlarged and dilated with gas, is precisely the same over the costal and the abdominal portion. This might seem to imply that we must regard the percussion note of the chest both in pitch and quality to be mainly dependent on the structures underneath. There are two answers to this objection. The first is that the tension of gas in the stomach is not necessarily that of the atmosphere. In fact, it is usually raised, and, as we have noted above, a pneumothorax with increased pressure raises the note considerably. The second is that this portion of the costal wall, unlike that which covers the lungs, hasa tension of its own, for it becomes lax and pliable as soon as the underlying structures are removed, despite the fact that a layer of pleura intervenes.

So far we have mainly been considering the question of pitch. It now behoves us to say a few words about the quality of the note. It will have been already observed that though the quality of the note on both subjects in our *post-mortem* experiment was apparently altered by death, the alteration was in no way changed by the evisceration of the thoracic contents. From our pot experiments we saw that the quality was altered when the pot was put rim downwards, and that it was altered again by stuffing the pot with cotton-wool. Whether this is due to the effect of cavity or whether to a damping of the vibrations it is difficult to say. Dr. Bullar shows us in his experimental pneumothorax that the loss of contact of the lung to the chest wall made practically no difference ; and we have shown that halving the cavity in the pot experiments, so long as the outer pot was free to vibrate, made no difference. Cases of pneumothorax occur which from physical signs are difficult to diagnose, and next to impossible to mark out by percussion. These are probably those in which, like Dr. Bullar's experimental pneumothorax, the air pressure in the cavity is unaltered. We would conclude then that we consider that the quality of the percussion note, like the pitch, is mainly due to the structure and tension of the chest wall, but that in disease it is secondarily due to changes in the chest cavity itself.

We shall conclude our paper with the following rider. In percussing a chest the resonance is considerably increased by making the patient stand up close to a door. Musical instruments are fashioned after two main types. There are those that depend for their note upon the rhythmical vibrations of air inside a tube, and there are those that make their note by the vibrations of a taut string or membrance. Time and experience have shown that it is the latter class whose resonance can be so immeasurably enhanced by a sounding-board or resonator.

### REFERENCES.

Bullar, J. F.—"On the Percussion of the Lungs and Chest," *St. Barth. Hosp. Rep.*, 1883, xix, 211.

Hutchison, R., and Rainy, H.—*Clinical Methods*, 4th Edition, 1908.

Gee, S.—*Auscultation and Percussion*, 5th Edition, 1906.

Le Fevre, E.—*Physical Diagnosis*, 2nd Edition, 1906.

Flint, A.—*A Manual of Auscultation and Percussion*, 1890.

